# Assigning ecological roles to the populations belonging to a phenanthrene-degrading bacterial consortium using omic approaches

**DOI:** 10.1371/journal.pone.0184505

**Published:** 2017-09-08

**Authors:** Sabrina Festa, Bibiana Marina Coppotelli, Laura Madueño, Claudia Lorena Loviso, Marianela Macchi, Ricardo Martin Neme Tauil, María Pía Valacco, Irma Susana Morelli

**Affiliations:** 1 Centro de Investigación y Desarrollo en Fermentaciones Industriales, CINDEFI (UNLP; CCT-La Plata, CONICET), La Plata, Argentina; 2 Centro Nacional Patagónico (CENPAT-CONICET), Puerto Madryn, Chubut, Argentina; 3 Centro de Estudios Químicos y Biológicos por Espectrometría de Masa- CEQUIBIEM, Facultad de Ciencias Exactas y Naturales, UBA, IQUIBICEN, CONICET; 4 Comisión de Investigaciones Científicas de la Provincia de Buenos Aires, La Plata, Argentina; MJP Rohilkhand University, INDIA

## Abstract

The present study describes the behavior of a natural phenanthrene-degrading consortium (CON), a synthetic consortium (constructed with isolated strains from CON) and an isolated strain form CON (*Sphingobium* sp. AM) in phenanthrene cultures to understand the interactions among the microorganisms present in the natural consortium during phenanthrene degradation as a sole carbon and energy source in liquid cultures. In the contaminant degradation assay, the defined consortium not only achieved a major phenanthrene degradation percentage (> 95%) but also showed a more efficient elimination of the intermediate metabolite. The opposite behavior occurred in the CON culture where the lowest phenanthrene degradation and the highest HNA accumulation were observed, which suggests the presence of positive and also negative interaction in CON. To consider the uncultured bacteria present in CON, a metagenomic library was constructed with total CON DNA. One of the resulting scaffolds (S1P3) was affiliated with the *Betaproteobacteria* class and resulted in a significant similarity with a genome fragment from *Burkholderia* sp. HB1 chromosome 1. A complete gene cluster, which is related to one of the lower pathways (meta-cleavage of catechol) involved in PAH degradation (ORF 31–43), mobile genetic elements and associated proteins, was found. These results suggest the presence of at least one other microorganism in CON besides *Sphingobium* sp. AM, which is capable of degrading PAH through the meta-cleavage pathway. *Burkholderiales* order was further found, along with *Sphingomonadales* order, by a metaproteomic approach, which indicated that both orders were metabolically active in CON. Our results show the presence of negative interactions between bacterial populations found in a natural consortium selected by enrichment techniques; moreover, the synthetic syntrophic processing chain with only one microorganism with the capability of degrading phenanthrene was more efficient in contaminant and intermediate metabolite degradation than a generalist strain (*Sphingobium* sp. AM).

## Introduction

A microbial consortium is defined as the natural association of two or more microbial populations of different species, which act together as a community in a complex system where everyone benefits from the activities of others [1]. To build a microbial consortium with the desired properties, the enrichment culture technique is the most accepted and powerful tool [2–5] and is able to adapt environmental communities to feedstocks under specific conditions with the expectation that the developing communities will be enriched with microbes showing the desired functions [6].

Due to the capability for complex metabolic interactions and inherent compartmentalization of the natural or engineered microbial consortia, they constitute a great promise in overcoming the limitations of single strain systems and prevent undesired cross-reactions and side products [7].

Presumably, consortia do not represent ‘symbiosis’ in a classical sense, but they are interpreted as syntrophic associations [8]. Syntrophy, meaning ‘feeding together’, is established as a thermodynamically interdependent and mutually beneficial metabolic process that occurs between organisms where the metabolism of a given compound by one organism only occurs when the end products are maintained at low concentrations via consumption by a second organism [9].

Natural microbial assemblages are often taxonomically highly complex and can encompass hundreds of different species [10]. Moreover, since most of the constituting lineages of naturally occurring communities are not available in pure cultures, natural microbial assemblages are difficult to reassemble and/or study under controlled conditions in the laboratory [8]. A shift from single-organism studies to whole community studies was made not only because a small fraction of the microorganisms present in a microbial community can be cultured but also because the behavior of microorganisms as pure cultures is different from their behavior in a microbial community [11]. A microbial consortium is a promising and simplified model for the study of multispecies mechanisms and interactions of consolidated bioprocessing and a platform for discovering efficient synergistic enzymes [12].

Now, omic approaches are used not only to identify the microbial diversity but also to understand the community composition and to assign an ecological role to the unculturable microbiota of a particular niche [13]. It is also useful to understand the functionality of microbial communities and the evolutionary processes that drive their dynamics [14].

Sequence data of metagenomics tell us “what is there” and “what it is capable of doing,” whereas what it is actually doing can be answered by proteomics. Proteomics-based investigations have been useful in determining changes in the composition and abundance of proteins and in the identification of key proteins involved in the physiological response of microorganisms when exposed to anthropogenic pollutants [15]. The combination of proteomics technology and classical scientific approaches will prove beneficial in determining the metabolic versatility of microorganisms used in bioremediation [16].

In previous work, a natural phenanthrene-degrading consortium (CON) was isolated from a chronically contaminated soil [17] and it was characterized in terms of diversity by pyrosequencing 16S rRNA gene fragments from its total DNA. This analysis revealed the presence of seven bacterial orders: *Sphingomonadales*, *Rhodospirillales*, *Rhizobiales*, *Xanthomonadales*, *Pseudomonadales*, *Enterobacteriales* and *Burkholderiales* [18,19]. Using culture-dependent methods, five strains were isolated that belonged to four of the seven mentioned orders: *Pseudomonadales*, *Enterobacterales*, *Rhodospirillales* and *Sphingomonadales*. The isolated strain identified as *Sphingobium* sp. (AM), which belongs to the latter order, was the only one with the ability to degrade phenanthrene as a sole carbon and energy source. Using a high-throughput technique, *Sphingobium* sp. AM`s draft genome was obtained and deposited in GenBank WGS (accession number LRUK00000000) [19].

The use of molecular methods and a combination of omic approaches would be helpful to understand the interaction among the strains present in the natural consortium. We used a metagenomic and metaproteomic approach to understand cultured and uncultured bacterial behavior in CON, and additionally, taking into account our previous knowledge, we designed a synthetic consortium (SC) with all of the strains isolated from CON to study the degrading potential and to compare it with the natural consortium.

## Materials and methods

### Isolation of a natural microbial consortium and construction of a synthetic consortium

From a chronically PAH-contaminated soil from Mosconi neighborhood (coordinates S 34° 52´31´ W 57° 55´10´) near La Plata City, Argentina (no specific permissions were required for this field location because it did not involve endangered or protected species), a phenanthrene-degrading bacterial consortium (CON) was obtained by culture enrichment technique in LMM with 2000 mg l^-1^ of phenanthrene as detailed in previous research [17].

Five different strains were isolated form CON using culture-dependent methods [17] and were used to construct a synthetic consortium (SC); these strains were *Sphingobium* sp. (AM), *Enterobacter* sp. (B), *Pseudomonas* sp. (T and Bc) and *Inquilinus limosus* (I). Each strain is grown individually in R2 liquid medium for 24 hours at 28°C at 150 rpm and then washed with saline solution and inoculated to form the SC, which was measured at 0.2 absorbance at 580 nm on day 0 of the treatment.

### Phenanthrene degradation studies

A degradation assay with CON, SC and *Sphingobium* sp. AM was conducted in triplicate in LMM supplemented with 200 mg.l^-1^ of sterile phenanthrene as a sole carbon and energy source. Cultures were incubated at 28°C, 150 rpm and monitored at 0, 4, 7 and 15 days. One non-inoculated Erlenmeyer was used as an abiotic control. The chemical extraction of phenanthrene and 1-hydroxy-2-naphthoic acid using ethyl acetate and the analysis of the organic extracts by high-pressure liquid chromatography (HPLC) using a Waters chromatograph with a Symmetry Waters C18 column and a diodearray detector were conducted as described in a previous work [17]. The statistical analysis of the phenanthrene degradation data were performed by a parametric one-way ANOVA test using the SigmaPlot/SigmaStat software program (SPSS Inc., Chicago, Illinois, USA).

### Microbial enumerations

Heterotrophic cultivable bacteria and PAH degraders were determined at 0, 4, 7 and 15 days of incubation. The determination of the first group was performed on R2A medium plates according to Reasoner and Geldreich [20], and determination of the latter was performed in sterile polypropylene microplates with a mixture of PAH as a substrate according to Wrenn and Venosa [21]. For a detailed protocol, see a previous work [17]. All of the determinations were performed in triplicate.

### Metagenomic library construction

#### DNA extraction

Total DNA was extracted from a culture of CON in LMM with 200 mg.l^-1^ of phenanthrene as a sole carbon and energy source after 2 days of incubation. The protocol was conducted as described by Entcheva and co-workers [22] with the aim of obtaining DNA fragments larger than 23 kb. The DNA concentration was measured using a NanoDrop 2000 (Thermo-Scientific^™^).

#### Metagenomic library construction and functional screening

The fosmid clone library was constructed using the CopyControl^™^ HTP Fosmid Library Production Kit with pCC2FOS^™^ Vector (Epicenter) according to the manufacturer’s recommendations. Briefly, fragments larger than 23 kb were selected and an end repair reaction was carried out to insert the ends, which were then ligated into the pCC2FOS^™^ vector, packed with MaxPlax Lambda Packaging Extracts and transfected into EPI300-T1R Phage T1-resistant *E*. *coli*. The resulting library was plated in LB medium containing 12.5 μg.ml^-1^ chloramphenicol, and approximately 8000 clones were replicated and analyzed by functional screening. The first functional screening was developed with an aqueous solution of catechol with a preceding exposure to phenol vapors for 6 h as described by our colleagues [23] with some modifications. Catechol-positive clones were submitted to a second functional screening using a 20 mg.ml^-1^ 2,3-dihydroxybiphenyl solution in acetone as detailed by Ren and co-workers [24]. Both screening methods were performed in LB medium diluted 1/10 with 12.5 μg.ml^-1^ chloramphenicol. The 18 isolated clones of 3 pools with 6 clones each were formed in order to sequence the insert they contained.

#### Fosmid extraction, sequencing, phylogenetic affiliation and gene annotation

The Fosmid extraction was conducted using a Fosmid MAX^™^ DNA Purification Kit (Epicenter) according to the manufacturer’s recommendations.

Fosmid DNA reads generated by Illumina and sequence assembling were performed at the Instituto de Agrobiotecnología Rosario (INDEAR, Argentina). Sequence data are available at the NCBI Sequence Read Archive (SRA) under accession number SRP108260. The phylogenetic affiliation of the fosmid insert was evaluated with PhylopythiaS software in the generic mode, which is a previously trained mode with publicly available prokaryotic genomes and a taxonomy available at NCBI and from compositional vectors [25]. Based on a structural support vector machine (SSVM) model, PhylopythiaS permits taxonomic assignment by analyzing the compositional vectors derived from DNA sequence fragments.

The open reading frames (ORFs) were identified and automatically annotated using de Rapid Annotation using Subsystem Technology (RAST, http://rast.nmpdr.org/). Automatically predicted genes were verified through a heuristic approach using FGENESB (http://linux1.softberry.com/berry.phtml). Final ORF annotations were performed with Artemis software by applying the criteria proposed by Pallejà and co-workers [26]. Putative functions of predicted ORFs were manually checked by BLASTp searches against all non-redundant protein sequences from the NCBI database and by functional analysis of deduced protein sequences using InterPro web service (http://www.ebi.ac.uk/interpro/), which allowed protein classification into families and predicted the presence of protein domains.

To classify RHO into functional classes and predict their potential substrates, the RHOBASE program was used [27].

#### Metaproteomic approach

Total protein extraction was performed with CON cultures in LMM supplied with 200 mg.l^-1^ of sterile phenanthrene as a sole carbon and energy source after 4 and 15 days of incubation (28°C, 150 rpm). The samples were filtered to discard the remaining phenanthrene crystals. Briefly, the extraction procedure included a first centrifugation for 10 minutes at 6000 rpm; consequently, the pellet was washed twice with MilliQ water and resuspended in MilliQ water to reach a final DO_580 nm_ of approximately 20. A protease inhibitor cocktail and 5 mM phenylmethylsulfonylfluoride (PMSF) protease inhibitor (freshly prepared) were added to each sample, and then the cells were disrupted using a Precellys 24 bead beater (Bertin Technologies) three times for 30 s until clarification. Finally, the samples were centrifuged for 20 minutes at 8000 rpm and the supernatant was retained. Three volumes of solubilization buffer was added (7 M urea, 2 M thiourea, 2% (w/v) Triton X-100, 65 mM DTT, 1% (w/v) and Amberlite 1% (w/v)), and the mixture was shaken for solubilization for 60 minutes. The protein concentration in the supernatant was determined using the Qubit fluorometer (Invitrogen).

Two-dimensional electrophoresis was conducted in a PROTEAN II xi 2-D (Bio-Rad, Hercules, CA) connected to a refrigeration bath II Multitemp (GE Healthcare) to detect differential protein expression levels (up- or down-regulated) in microorganisms present in CON. The gels were calibrated with a molecular mass marker PageRuler^™^ Prestained Protein Ladder (10–170 kDa) (Pierce Endogen), and the spots were visualized with Coomasie Blue G250 stain and documented with Universal Hood II (Bio-Rad, Hercules, CA).

Image analysis, including spot detection, matching, abundance quantification, and normalization, were performed using ProteomeWeaver software (Definiens, Munich, Alemania). Each analyzed condition of CON was evaluated in duplicate as independent cultures.

The spots of interest were manually excised and sent to the Mass Spectrometry Facility (CEQUIBIEM) at the School of Exact and Natural Sciences, University of Buenos Aires. Tryptic in-gel digestion was performed, and extracted peptides were analyzed in a UV-MALDI-TOF/TOF (UltraflexII BrukerDaltonics). The software used for spectrum visualization and MS/MSMS protein identification was Flex Analysis (v. 3.3) and BioTools (Bruker Daltonics) linked to MASCOT (Matrix Science, Boston, MA 2016 (http://www.matrixscience.com/) to search against the NCBInr protein sequence databases.

## Results

### Phenanthrene degradation by the synthetic consortium compared to CON and AM strain

**Fig 1:**
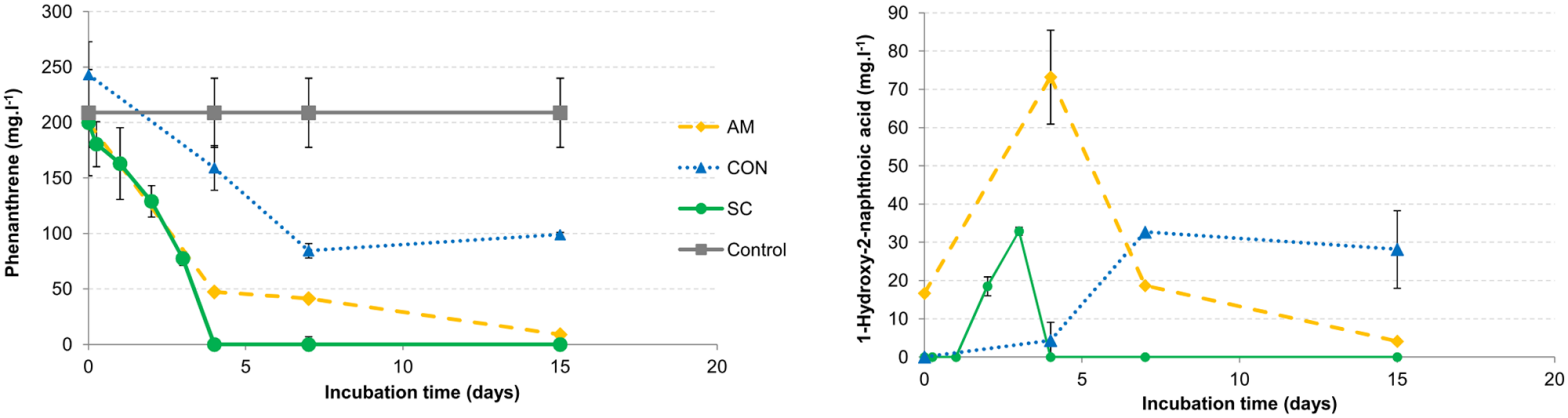
Concentration of phenanthrene and 1-hydroxy-2-naphthoic acid (HNA) in AM, CON and SC in phenanthrene-supplemented cultures. The (A) phenanthrene and (B) 1-hydroxy-2-naphthoic acid (HNA) concentrations in AM, CON and SC cultures growing in LMM with phenanthrene as a sole carbon and energy source during 15 days of incubation. The results are the means of triplicate independent experiments. The bars represent standard deviations.

The degradation efficiency of CON, AM strain and the synthetic consortium (SC) was studied in LMM with 200 mg.l^-1^ of phenanthrene as the only carbon and energy source and is shown in Fig 1. After 4 days of incubation of AM strain and SC cultures, the residual concentration of phenanthrene was significantly lower than in CON, whereas after 4 days of incubation, CON degraded 34% of the phenanthrene supplied and AM strain and SC degraded more than 75% and 99%, respectively.

At the end of the incubation time (15 days) in CON cultures, the phenanthrene concentration remained constant, whereas in AM and SC, it decreased until more than 95% of the phenanthrene was eliminated. During phenanthrene degradation, the HNA accumulation was monitored (Fig 1B). In CON cultures, the HNA concentration increased during the first 7 days of incubation and then it remained constant because it was the culture that showed a significantly higher concentration of HNA at the end of the incubation time. In the cultures of AM strain and SC, the maximum HNA accumulation occurred early (during the first 4 days of incubation); later on, the HNA concentration in SC decreased rapidly (not detected after day 5), whereas in AM cultures, it decreased gradually until day 14.

**Fig 2:**
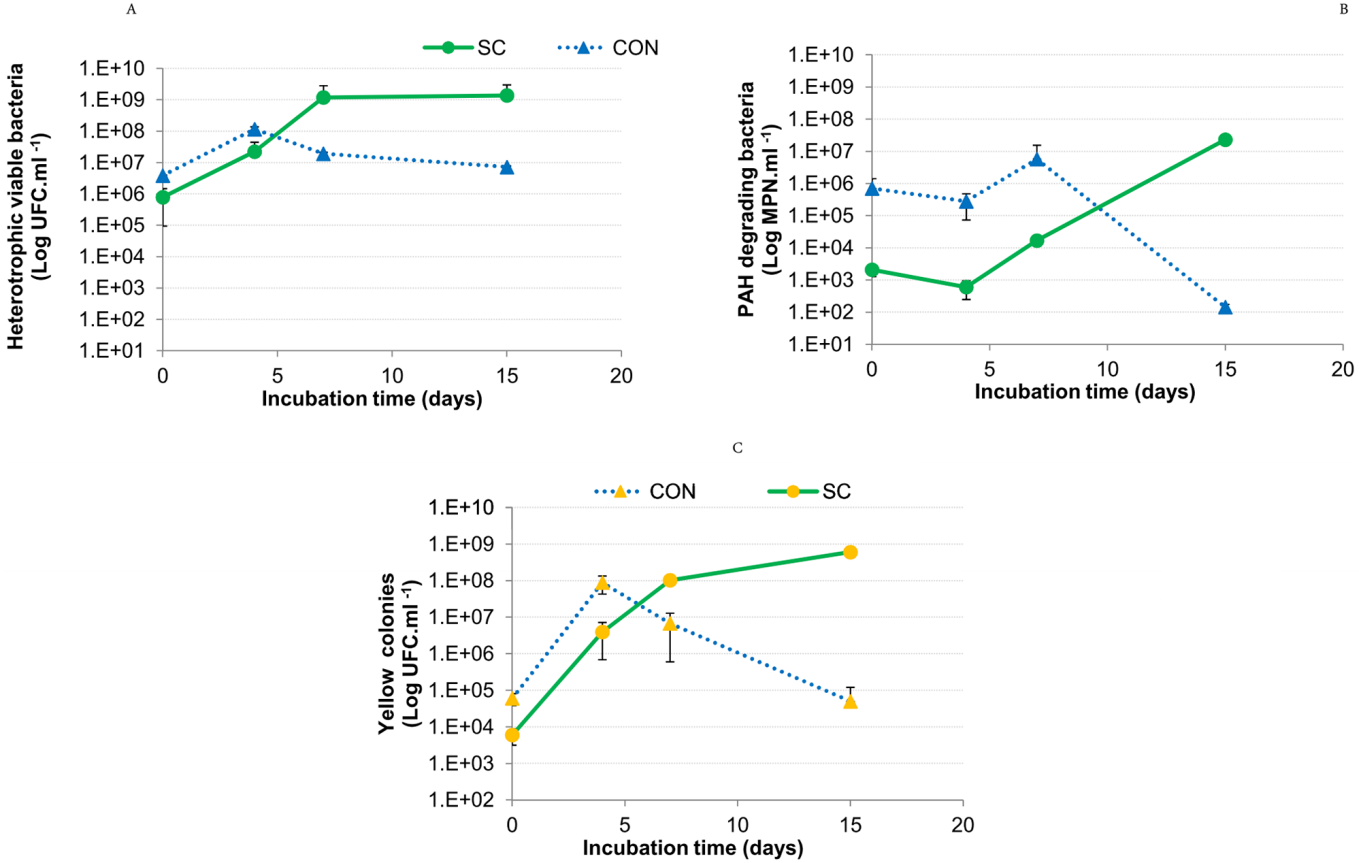
Bacterial counts in CON and SC cultures in phenanthrene-supplemented cultures. Heterotrophic viable bacteria (A) and PAH-degrading bacteria (B) counts in CON and SC cultures growing in LMM with phenanthrene as a sole carbon and energy source during 15 days of incubation. Yellow colony (YC) counts in CON and SC during phenanthrene degradation (C). The results are the means of triplicate independent experiments. The bars represent standard deviations.

The dynamic of the different populations present in CON and SC during phenanthrene degradation is shown in Fig 2A. In CON, the heterotrophic population increased up to 10^8^ CFU.ml^-1^ until day 5 of incubation and then declined to 10^6^ CFU.ml^-1^. On the contrary, in SC, the heterotrophic viable bacteria increased its value until the end of the assay and achieved 10^9^ CFU.ml^-1^, which was significantly higher than CON (more than 3 orders of magnitude). With regard to the behavior of PAH-degrading bacteria, it was significantly different for both consortia. For instance, in CON, the PAH-degrading bacteria increased during the first 7 days of incubation and then declined robustly, whereas PAH-degrading bacteria in SC declined during the first four days of incubation followed by a significant increase that reached 5 orders of magnitude higher than in CON.

To compare the cultivable yellow colonies (within which is the AM strain) behavior in CON and in the synthetic consortium, a differential count was performed (Fig 2B). The counts of the yellow colonies showed the same behavior as the PAH-degrading bacteria, whereas in CON, the yellow colony count value diminished after 4 days of incubation and in SC, it increased until the end of the assay and was significantly higher than in CON from day 7 until the end of the incubation period.

### Metagenomic approach in CON culture

A metagenomic library was constructed with DNA recovered from a culture of CON growing in phenanthrene as a sole carbon and energy source after 2 days of incubation and using a fosmid as a vector. The 8000 obtained clones covered approximately 69 Mb of total genomic DNA of CON. To find the oxygenase activity involved in the degradation pathway of aromatic compounds, a functional screening was performed using catechol and 2,3-hydroxybiphenyl as a substrate, and both enzymatic products are evidenced by a yellow color in the colony. As a result of function-based screening, 18 clones of interest were selected with catechol and 9 of them were also selected using 2,3-hydroxybiphenyl. Sequencing yielded 10 scaffolds, and only 3 of the 10 resulting assembled scaffolds showed a high number of genes codifying proteins directly involved in PAH degradation (99% identity among the 3 scaffolds). In addition, in these three scaffolds, the genes linked to the bacterial response to the presence of hydrocarbon and mobile genetic elements and associated proteins were also found (Table 1).

**Table 1:** Functional assignment of the identified coding sequences found in S1P3.

CDS	Function
1	3-phenylpropionate dioxygenase, alpha subunit (EC 1.14.12.19)
2	2-hydroxychromene-2-carboxylate isomerase family protein, glutathione-dependent
3	hypothetical protein
4	hypothetical protein
5	hypothetical protein
6	hypothetical protein
7	Positive regulator of phenol hydroxylase
8	hypothetical protein
9	hypothetical protein
10	hypothetical protein
11	Nitrilotriacetate monooxygenase component A (EC 1.14.13.-)
12	hypothetical protein
13	2-dehydropantoate 2-reductase (EC 1.1.1.169)
14	4-hydroxy-tetrahydrodipicolinate synthase (EC 4.3.3.7)
15	hypothetical protein
16	4-hydroxythreonine-4-phosphate dehydrogenase (EC 1.1.1.262)
17	Benzoate 1,2-dioxygenase beta subunit (EC 1.14.12.10)
18	Benzoate 1,2-dioxygenase alpha subunit (EC 1.14.12.10)
19	Choline dehydrogenase (EC 1.1.99.1)
20	Ortho-halobenzoate 1,2-dioxygenase beta-ISP protein OhbA
21	Large subunit naph/bph dioxygenase
22	Long-chain fatty acid transport protein
23	4-hydroxy-tetrahydrodipicolinate synthase (EC 4.3.3.7)
24	Toluene-4-monooxygenase, subunit TmoF
25	2-hydroxy-6-oxo-6-phenylhexa-2,4-dienoate hydrolase (EC 3.7.1.-)
26	luciferase family protein
27	Ferredoxin subunits of nitrite reductase and ring-hydroxylating dioxygenases
28	2,3-dihydroxybiphenyl 1,2-dioxygenase (EC 1.13.11.39)
29	Quinone oxidoreductase (EC 1.6.5.5)
30	2-hydroxychromene-2-carboxylate isomerase
31	Large subunit toluate/benzoate dioxygenase
32	Ortho-halobenzoate 1,2-dioxygenase beta-ISP protein OhbA
33	1,2-dihydroxycyclohexa-3,5-diene-1-carboxylate dehydrogenase (EC 1.3.1.25)
34	2-hydroxymuconic semialdehyde hydrolase (EC 3.7.1.9)
35	Glutathione S-transferase (EC 2.5.1.18)
36	4-oxalocrotonate tautomerase (EC 5.3.2.-)
37	4-oxalocrotonate decarboxylase (EC 4.1.1.77)
38	4-hydroxy-2-oxovalerate aldolase (EC 4.1.3.39)
39	Acetaldehyde dehydrogenase, acetylating, (EC 1.2.1.10) in gene cluster for degradation of phenols, cresols, catechol
40	4-oxalocrotonate decarboxylase (EC 4.1.1.77)
41	Putative 5-carboxymethyl-2-hydroxymuconate semialdehyde dehydrogenase oxidoreductase protein (EC 1.2.1.60)
42	Catechol 2,3-dioxygenase (EC 1.13.11.2)
43	Iron-sulfur binding electron transfer protein
44	hypothetical protein
45	hypothetical protein
46	hypothetical protein
47	Regulatory protein GntR, HTH:GntR, C-terminal
48	Mobile element protein
49	Mobile element protein
50	4-hydroxybenzoate transporter
51	Outer membrane protein (porin)
52	Mobile element protein
53	Mobile element protein
54	Mobile element protein
55	Mobile element protein
56	hypothetical protein
57	Integrase/recombinase
58	Integrase/recombinase
59	Integrase/recombinase
60	Mobile element protein
61	Mobile element protein
62	hypothetical protein
63	Mobile element protein
64	Mobile element protein
65	Mobile element protein
66	Mobile element protein
67	Conjugal transfer protein traA
68	Hyphotheical protein

As expected, genes coding 2,3-hydroxybiphenyl 1,2-dioxygenase and catechol 2,3-dioxygenase were found as a result of the selected substrates used for functional screening.

#### Scaffold 1 pool 3 (S1P3) phylogenetic affiliation

The G+C content of S1P3 was 54.8%. Because no rRNA genes were found in this fragment, the microbial origin of the fosmid insert was analyzed with PhyloPhythiaS software [25], which is a phylogenetic classifier based on oligonucleotide composition. The results showed that S1P3 was related to *Proteobacteria* affiliated with the *Betaproteobacteria* class. Moreover, the comparison between this insert and the NCBI genome database using the BLASTn program of BLAST resulted in a significant similarity with a genome fragment from *Burkholderia* sp. HB1 chromosome 1; both sequences shared 99% identity (a query coverage of 80%).

#### Gene prediction of S1P3

A total of 68 ORFs were identified in this fragment. For detailed information of the putative functions and references to the best BLASTp hits used for annotation, see the supporting information in S1 Table. The genetic organization and gene orientation of 27 ORFs were identical to that observed in the genomic fragment of *Burkholderia* sp. HB-1. Moreover, the predicted proteins from these 27 ORFs showed high coverage (except ORFs 20 and 24) and shared 97–100% homology at the amino-acid level with respect to corresponding proteins from *Burkholderia* sp. HB-1 (S2 Table).

#### Key enzymes involved in PAH degradation and putative substrates

**Fig 3:**
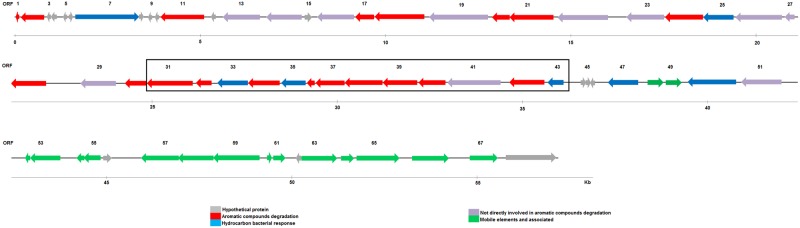
Gene organization of S1P3 fragment. The predicted coding sequences are shown with arrows and are colored by groups: genes codifying for hypothetical proteins (gray), genes codifying for aromatic compound degradation (red), genes codifying for the hydrocarbon bacterial response (light blue), genes not directly involved in aromatic compound degradation proteins (violet) and genes for mobile elements and associated proteins (green). Coding sequences are numerated every three. The black box highlights a complete gene cluster with ORFs related to a lower pathway involved in PAH degradation.

From the ORFs identified in S1P3, 38% corresponded to putative genes involved in PAH biodegradation. The gene organization is presented in Fig 3. Among these genes, the genes encoding alpha and beta subunits of ring hydroxylating oxygenases (RHO, or Rieske non-heme iron oxygenases) (ORFs: 17, 18, 20, 21, 31, 32) (Table 1) and ring cleavage dioxygenases (ORFs: 28, 42), which are key enzymes for the diversification of metabolic pathways in PAH degradation, were found. The prediction of RHO putative substrates with the bacterial Ring-Hydroxylanting Oxigenase database revealed that ORF 18 could belong to a dioxygenase class B and that carboxylated compounds like benzoate and toluate are its potential substrates. Deduced gene products of ORFs 21 and 31 were classified as oxigenases class C, which are enzymes with a preference for carboxylated aromatics like salicylates, anthranilate and dihydroxybenzoates. Although ORF 01 corresponds to a partial sequence encoding a putative oxygenase, it was also analyzed with RHObase. Furthermore, it was classified as RHO class A and possible substrates were low-molecular-weight polycyclic aromatic hydrocarbons like naphtalene and phenanthrene and hetero polycyclic hydrocarbons like dibenzothiophene sulfone. Potential substrates of ring cleavage dioxygenases encoded by ORFs 28 and 42 were predicted using an AromaDeg web-based resource. This prediction enables one to infer that ORFs 28 and 42 gene products were extradiol dioxigenases (EXDO) involved in bicyclic and monocyclic substrate cleavage, respectively.

#### Putative genes involved in the upper and lower pathways of PAH degradation

Four ORFs (02, 23, 25 and 30), which are probably involved in the upper pathway of PAH biodegradation, were also detected. ORFs 02 and 30 shared 99 and 98%, respectively, identity at the amino acid level with two isomerases from *Burkholderia* genus (S2 Table). Both protein sequences showed domains corresponding to the 2-hydroxychromene-2-carboxylate (HCCA) isomerase subfamily, and the fourth enzyme in the six-step pathway converts napthalene into salicylate. Protein sequences from ORFs 23 and 25 were also related to *Burkholderia* genus (S1 Table) and presented high homology (100 and 99% identity), respectively, with an aldolase and a putative hydrolase (S2 Table). ORF 23 contained a protein domain that belonged to the dihydropicolinate synthase family with trans-o-hydroxybenzylidene pyruvate hydratase-aldolase as one of its members. However, the prediction function of ORF 25 revealed that this sequence could encode a 2-hydroxy-6-oxo-6-phenylhexa-2,4-dienoate hydrolase, which is the fourth enzyme involved in biphenyl biodegradation.

A complete gene cluster with ORFs related to one of the lower pathways involved in PAH degradation was identified in a region of 11.8 kb (ORFs 31–43) (Fig 3). It comprises sequences coding for the first to the last enzyme implicated in the conversion of catechol to acetyl CoA: a catechol 2.3-dioxygenase (ORF 42), 2-hydroxymuconic semialdehyde hydrolase (ORF 34), 2-hydroxymuconic semialdehyde dehydrogenase (ORF 41), 4-oxalocrotonate tautomerase (ORF 36), 4-oxalocrotonate decarboxylase (ORF 37), enoate hydratase (40), 4-hydroxy-2-oxovalerate aldolase (ORF 38) and acetaldehyde dehydrogenase (ORF 39). Another ORF detected in this cluster corresponded to a 1,2-dihydroxycyclohexa-3,5-diene-1-carboxylate dehydrogenase (ORF 33), which is an enzyme that produces catechol from 1,2-dihydroxycyclohexa-3,5-diene-1-carboxylate during benzoate degradation. An ORF related to a glutathione S- transferase (GST) was also identified in this operon (ORF 35).

**Fig 4:**
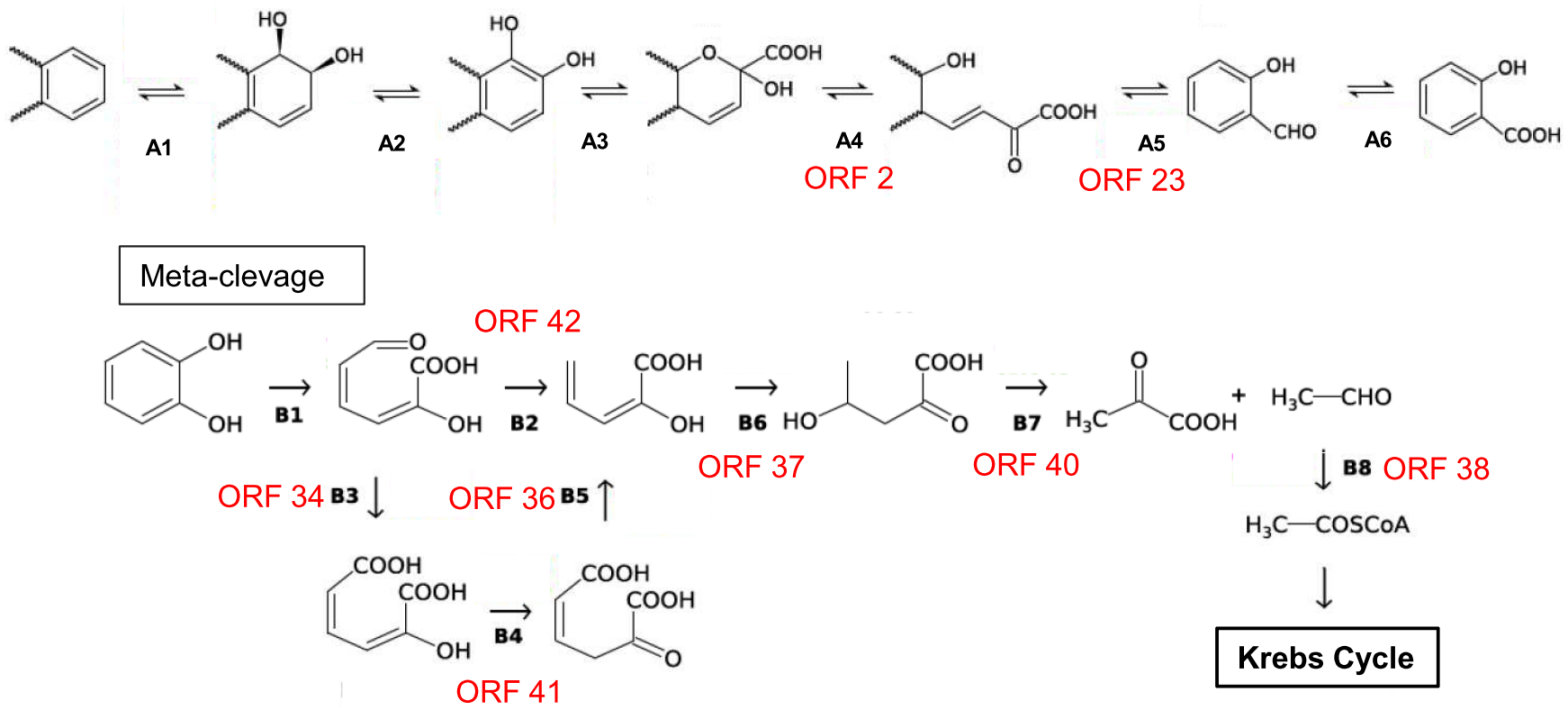
Metabolic reactions that could involve proteins codified in the ORFs found in S1P3. (1) The upper degradation pathway of aromatic compounds: A1 to A6, which were initial dioxygenase, dehydrogenase, extradiol dioxygenase, isomerase, hydratase/aldolase and aldehyde dehydrogenase, respectively. (2) The lower degradation pathway (meta-cleavage of catechol): B1 to B8, which were catechol 2, 3-dioxygenase, hydroxymuconic- semialdehyde dehydrogenase, hydroxymuconic-semialdehyde hydrolase, 4-oxalocrotonate isomerase, 4-oxalocrotonate decarboxylase, 2-keto-4-pentenoate hydratase, 2-oxo-4-hydroxypentenoate aldolase and acetaldehyde dehydrogenase, respectively.

The reactions in the upper and lower pathways of PAH degradation that could be catalyzed by these enzymes are shown in Fig 4.

Although a gene that encodes a monooxygenase enzyme was identified in the fosmid insert (ORF 11), the homology analysis with BLASTp and the prediction of conserved domains using InterPro showed that ORF 11 is not related to monooxygenases involved in phenol activation or similar compounds such as toluene or benzene. The gene products of ORF 11 and ORF 26 seem to be members of the luciferase-like monooxygenase family.

#### Regulation, substrate transport and mobile elements

ORF 47, which encodes a transcriptional regulator related to GntR proteins, was detected upstream of the catechol 2,3-dioxygenase gene preceded by a ferredoxin and followed by 2-hydroxymuconic semialdehyde dehydrogenase. However, ORF 7 encoded a regulatory protein (Fig 3 and Table 1) and was associated with a positive regulator of phenol hydroxylase.

Genes encoding membrane proteins related to substrate transport (ORFs 50 and 51) were detected upstream of the catabolic genes. In particular, ORF 50 encoded a 4-hydroxybenzoate transporter (Table 1).

S1P3 was characterized by a relative abundance in genes associated with mobile element proteins and their remnants at the end of the DNA fragment (ORFs 48, 49, 52–55, 57–61 and 63–66; Fig 3 and Table 1).

#### PAH degradation pathway enzymes found in *Sphingobium* sp. AM strain genome

Different enzymes involved in the diverse metabolic steps in PAH degradation pathway were found in the assembled draft genome of *Sphingobium* sp. AM strain [19], which allows AM strain to follow either meta- or ortho-cleavage pathway for phenanthrene degradation, and these include all enzymes participating in the steps shown in Fig 4. All of the dioxygenase enzymes found in the genome of *Sphingobium* sp. AM strain are detailed in Table 2, in particular, the dioxygenase enzyme could be involved in the A1 and A3 and B1 metabolic steps of Fig 4. Using RHObase, it was determined that in *Sphingobium* sp. AM strain, the aromatic ring hidroxylating dioxygenase (present in four copies) belongs to class A, the benzoate 1,2 dioxygenase belongs to class B, the toluate/benzoate dioxygenase/ BphA1d and Rieske (2Fe-2S) proteins belong to class C, and Salicylate 1-monooxygenase and 2Fe-2S ferredoxin belong to class D (Table 2). *Sphingobium* sp. AM shared some of the metabolic steps with the scaffold S1P3, and most of them are involved in the PAH lower degradation pathway (Fig 4).

**Table 2:** Dioxygenase enzymes codified in the genome of *Sphingobium* sp. AM strain and its putative substrates.

Dioxygenase enzymes codified in the genome of *Sphingobium* sp. AM strain	ORFs	Putative oxygenase	RHO base analysis—putative substrates
Aromatic-ring-hydroxylating dioxygenase alpha and beta subunits	4	A	Polycyclic aromatic hydrocarbons like Naphthalene, Phenanthrene, and Anthracene, among others
Benzoate 1,2-dioxygenase alpha and beta subunits	1	B	Carboxylated aromatics like Benzoate and Toluate
2,3-dihydroxybiphenyl 1,2-dioxygenase	1	No hits found	
Toluate 1,2-dioxygenase subunit alpha	1	C	Carboxylated aromatics like Salicylate and Anthranilate
Biphenyl 2,3-dioxygenase	1	No hits found	
Anthranilate 1,2-dioxygenase beta subunit	2	No hits found	
Salicylate 1-monooxygenase	1	D	Hetero polycyclic hydrocarbons like Carbazole, Chlorinated dibenzofurans, Diphenylamine and 2-Oxo-1,2-dihydroquinoline
Homogentisate 1,2-dioxygenase	1	No hits found	
Catechol 2,3-dioxygenase	2	No hits found	
Catechol 1,2-dioxygenase	2	No hits found	
Rieske (2Fe-2S) protein	1	C	Carboxylated aromatics like Salicylate and Anthranilate
2Fe-2S ferredoxin	10	D	Carboxylated aromatics like Phthalate, Chlorobenzoate, Methoxy dichlorobenzoate, Toluene-4-sulfonate, and Vanillate Phenoxybenzoate and Mono- and Di-chlorophenoxybenzoates and Hetero polycyclic hydrocarbons like Chlorinated dibenzofurans

#### Metaproteomic approach

A comparative 2-DE analysis of CON was performed to analyze the protein expression profile at different times during phenanthrene degradation in LMM supplemented with 200 mg.l^-1^ of phenanthrene as a sole carbon and energy source.

**Fig 5:**
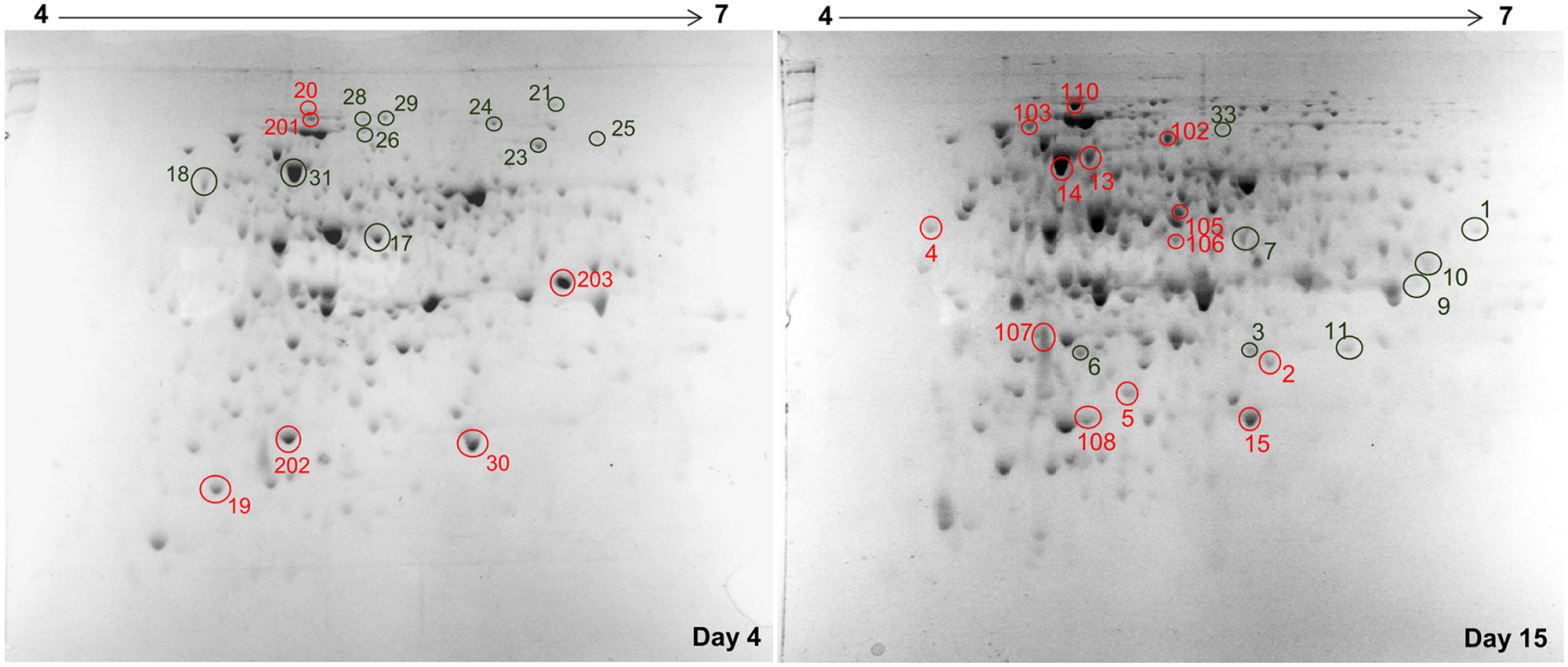
2-DE electrophoresis gels of the soluble fractions of proteins extracted from CON after 4 and 15 days of incubation. The marked spots were analyzed by MALDI TOF/TOF MS/MS. The identified spots are shown in red, and the ones with a low score shown in green were unidentified. The numbers match with the numbers in Table 2.

The soluble protein fractions were analyzed by two-dimensional electrophoresis in a pH range of 4–7 and a molecular weight range of 6.5–200 kDa. As a result, approximately 200 spots were detected in each gel image (Fig 5), and the gel of day 15 was the one with the higher number of spots. Through an image analysis of the gel of day 4, 199 spots were detected, of which 62 were over-expressed in comparison to day 15. Through an image analysis of the gel of day 15, 231 spots were detected, of which 94 were over-expressed in comparison to day 4. From both gels, 41 spots were chosen as differentially expressed in both incubation times or for being in the same position in both conditions and to confirm the same identity. After a Mascot search using mass spectra data generated by peptide mass fingerprinting and MSMS, 51% of the proteins were identified, 67% of which belong to *Alphaproteobacteria* class, *Sphingomonadaceae* family (*Sphingobium* and *Sphingomonas* genera) and 33% to *Betaproteobacteria*, *Burkholderiaceae* family (*Burkholderia* and *Achromobacter* genera). The proteins identified from the latter family were only observed at day 15. The database search results are shown in Table 3, which includes the unidentified proteins (low score).

**Table 3:** Mascot results of identified and non-identified proteins found in 2-DE electrophoresis gels of CON cultures. Summary of the Mascot search results of identified (shaded grey) (high score and/or high sequence coverage) and non-identified proteins (low score) found in 2-DE electrophoresis gels of CON cultures after 4 and 15 days of incubation and analyzed by MALDI TOF/TOF MS/MS. The “x” indicates proteins present in each condition. The lower the expectation value (e-value), the more significant is the score.

Spot ID	Protein description	Day		Protein accession	Microorganism	Mass (Da)	Protein Score	Score >	e-value	Order	AM
		**4**	**15**								
2	peroxidase		x	gi|913648761	Achromobacter piechaudii	23911	108	87	0.0004	Burkholderiales	
4	flagellar motor protein MotB		x	gi|503612114	Sphingobium chlorophenolicum	37434	102	87	0.0017	Sphingomonadales	x
5	hemerythrin	x	x	gi|739609734	Sphingobium quisquiliarum	19421	234	90	2E-16	Sphingomonadales	x
13	MULTISPECIES: molecular chaperone GroEL		x	gi|493430365	Achromobacter	57416	190	87	2.7E-12	Burkholderiales	
14	molecular chaperone GroEL	x	x	gi|493268672	Sphingobium chlorophenolicum	57331	194	90	0.00017	Sphingomonadales	x
15	MULTISPECIES: glutathione S-transferase	x	x	gi|158346886	Sphingomonadaceae family	21470	234	90	2E-16	Sphingomonadales	x
19	MULTISPECIES: anthranilate 1,2-dioxygenase small subunit	x	x	gi|496103626	Sphingomonadaceae family	18116	185	86	7.7E-12	Sphingomonadales	x
30	MULTISPECIES: glutathione S-transferase	x	x	gi|158346886	Sphingomonadaceae family	21470	191	87	2.1E-12	Sphingomonadales	x
100	OmpA/MotB domain protein		x	gi|307294285	Sphingobium chlorophenolicum L-1	37434	96	81	0.002	Sphingomonadales	
102	acid-coenzyme A ligase		x	gi|317402668	Achromobacter xylosoxidans C54	60191	93	81	0.004	Burkholderiales	
103	chaperone protein DnaK		x	gi|311104375	Achromobacter xylosoxidans A8	69572	103	81	0.00038	Burkholderiales	
105	seryl-tRNA synthetase		x	gi|307294312	Sphingobium chlorophenolicum L-1	46894	105	81	0.00024	Sphingomonadales	x
106	translation elongation factor Tu 1		x	gi|311109614	Achromobacter xylosoxidans A8	42919	105	81	0.00024	Burkholderiales	
107	10 kDa chaperonin, GroES		x	gi|94497511	Sphingomonas sp. SKA58	10252	91	81	0.0055	Sphingomonadales	x
108	benzene dioxygenase small subunit		x	gi|158346882	Sphingomonas sp. LH128	18996	191	81	6E-13	Sphingomonadales	x
110	aconitate hydratase 1	x	x	gi|307293228	Sphingobium chlorophenolicum L-1	96376	104	81	0.0003	Sphingomonadales	x
111	chaperonin		x	gi|317405440	Achromobacter xylosoxidans C54	57416	138	81	1.2E-07	Burkholderiales	
113	putative oxidoreductase		x	gi|53720358	Burkholderia pseudomallei K96243	23904	90	81	0.0081	Burkholderiales	
201	aconitate hydratase 1	x	x	gi|307293228	Sphingobium chlorophenolicum L-1	96376	167	81	1.5E-10	Sphingomonadales	x
202	10 kDa chaperonin, GroES	x		gi|94497511	Sphingomonas sp. SKA58	10252	94	81	0.003	Sphingomonadales	x
203	hypothetical protein SJA_C1-23830	x		gi|294012369	Sphingobium japonicum UT26S	36651	284	84	5.2E-22	Sphingomonadales	x
1	ABC transporter substrate-binding protein		x	gi|916882109	Roseivivax halodurans	38117	63	87	13	Burkholderiales	
3	electron transfer flavoprotein subunit beta	x	x	gi|544831343	Sphingobium baderi	26340	59	86	33	Sphingomonadales	
6	MULTISPECIES: hypothetical protein		x	gi|490369075	Enterobacteriaceae	10525	70	87	2.9	Enterobacteriales	
7	Elongation Factor Tu, partial	x	x	gi|399117417	Taylorella asinigenitalis 14/45	17172	61	87	11	Burkholderiales	
8	glycine—tRNA ligase subunit beta	x	x	gi|917070028	Sphingomonas astaxanthinifaciens	73368	86	87	220	Sphingomonadales	
9	MULTISPECIES: glutamate dehydrogenase	x	x	gi|757782327	Gammaproteobacteria	49024	66	87	27	Enterobacteriales	
10	hypothetical protein		x	gi|504827049	Acidovorax sp. KKS102	11788	68	87	3.8	Burkholderiales	
11	PKHD-type hydroxylase		x	gi|759428465	Sphingobium sp. Ant17	24354	79	87	12000	Sphingomonadales	x
17	L-ascorbate-6-phosphate lactonase ulaG	x		gi|595625006	Klebsiella pneumoniae 30684/NJST258_2	41773	55	87	94	Enterobacteriales	
18	transcriptional regulator	x		gi|565806306	Xanthomonas hortorum	30827	60	87	27	Xanthomonadales	
20	DNA gyrase subunit A	x		gi|670491751	Sphingomonas sp. FUKUSWIS1	101085	64	87	12	Sphingomonadales	
21	type I restriction modification DNA specificity domain protein	x		gi|805605932	Burkholderia pseudomallei MSHR1079	48418	67	87	5.5	Burkholderiales	
23	transposase	x		gi|852159985	Klebsiella pneumoniae	8434	64	87	10	Enterobacteriales	
24	protein disulfide-isomerase	x		gi|916789948	Bradyrhizobium elkanii	23273	72	87	72	Rhizobiales	
25	plasmid partitioning protein RepB	x		gi|516731572	Rhizobium leguminosarum	35961	58	87	42	Rhizobiales	
26	hypothetical protein SEEE6482_13432	x		gi|396069711	Salmonella enterica	12520	51	87	220	Enterobacteriales	
28	hypothetical protein M770_18930	x		gi|532132637	Pseudomonas aeruginosa VRFPA03	16995	74	87	1.2	Pseudomonadales	
29	MULTISPECIES: cysteine ABC transporter substrate-binding protein	x		gi|970415048	Enterobacter	29569	65	87	8.4	Enterobacteriales	
31	hypothetical protein OO17_23135	x	x	gi|763444705	Rhodopseudomonas palustris	8507	81	87	0.24	Rhizobiales	
33	MULTISPECIES: (Fe-S)-cluster assembly protein		x	gi|760372574	Pseudomonas	11704	57	87	51	Pseudomonadales	

With regard to the identified proteins, glutathione S-transferase (spots 15 and 30), chaperone GroEL (spot 14), GroES (spots 107 and 202) and Aconitate Hydratase 1 (spots 110 and 201) were found in both analyzed conditions together with an anthranilate 1,2-dioxygenase small subunit (shared spot: 19). A toluate/benzoate dioxygenase small subunit (XylY) was only present at day 15.

Considering the unidentified proteins, only an electron transfer flavoprotein subunit beta (belonging to *Sphingomonadales*) (spot 3), a PKHD-type hydroxylase (belonging to *Sphingomonadales*) (spot 11), an (Fe-S)-cluster assembly protein (belonging to *Pseudomonadales*) (spot 33), which were all found at day 15, could be involved in PAH degradation.

Even though not all of the analyzed proteins were identified, all of the analyzed spots belonged to any genera present in CON.

Almost all of the identified proteins belonging to *Sphingomonadales* order were found to be codified in *Sphingobium*. sp AM genome (Table 3). On the contrary, none of the proteins belonging to the *Burkholderiales* order were codified in S1P3.

Spots 19 and 108 were analyzed with RHObase. Spot 19, which was identified as anthranilate 1,2-dioxygenase small subunit, was classified in RHO as a dioxygenase class C (whose possible substrates are carboxylated aromatics like salicylate and anthranilate), and spot 108 was classified in RHO as a dioxygenase class B (whose possible substrates are carboxylated aromatics like benzoate and toluate).

## Discussion

Microorganisms in the environment interact and communicate with one another as a dynamically changing microbial community [11].

Even a simple assemblage of two microbial genotypes can exhibit surprisingly complex and unexpected dynamics, which result in community-level functionalities and behaviors (e.g., robustness, resilience, complementarity, facilitation, competition, and antagonism) that might not be readily expected from analyzing each genotype in isolation. The understanding of the rules and principles that govern the dynamics and emergent functionalities of microbial assemblages is in its infancy [28].

In the present study, two different omic approaches were conducted to understand the interaction among several strains present in a previously obtained natural consortium (CON) [17]. Also, a synthetic consortium was constructed and phenanthrene degradation and microbial counts were compared to those obtained in CON.

After 15 days of incubation, degradation of the supplied phenanthrene was higher in the synthetic consortium (SC) than in AM strain and in CON (Fig 1A), and CON was the one with the lower PAH elimination. A similar result regarding a synthetic consortium and an isolated strain was previously reported [29,30]. Wang and co-workers [30] designed a consortium (W4) capable of aerobic biodegradation of phenanthrene formed by *Sphingomonas*, *Rhizobium*, *Pseudomonas* and *Achromobacter* genera. W4 showed a significant improvement in phenanthrene degradation in comparison to W4-1 strain (*Sphingomonas cloacae)*, which was the only strain capable of metabolizing the mentioned PAH. In this study, the major phenanthrene degradation percentage in the SC culture was accompanied by a higher number in the heterotrophic cultivable bacteria at the end of the assay (Fig 2A). Furthermore, PAH-degrading bacteria (Fig 2B) and yellow colonies did not decrease as was observed in the natural consortium (Fig 2C). However, HNA accumulation was lower in AM than in CON and was not detected in SC cultures (Fig 1B), which indicated that the presence of *Pseudomonas*, *Enterobacter* and *Inquilinus* could contribute to metabolite elimination. These results may suggest the presence of positive interactions (that allow the elimination of intermediate metabolites) but also negative interactions (that cause lower phenanthrene degradation in comparison with AM strain and SC) between bacterial populations present in CON.

Syntrophic interactions, where species consume metabolites excreted by others, are common in microbial communities [31]. However competition occurs when two species use the same resource; competitive exclusion precludes two populations from occupying exactly the same niche because one will win the competition and the other will be eliminated [32]. In cultures of CON in LMM supplemented with phenanthrene, after 4 days of incubation, the yellow colony counts showed a decrease in the number of AM strain (Fig 2C). It has been suggested that this elimination occurs rapidly in spatially homogeneous batch cultures [33]. Interspecific competition has been shown to be an important ecological mechanism for the evolution of cooperation within species [34]. The phenanthrene degradation assay (Fig 1A) and the analysis of the assembled draft genome of *Sphingobium* sp. AM strain (Fig 4) revealed that this strain could be considered a single generalist and a species that can fully metabolize the resource. It is not possible for a generalist to coexist with an entire processing chain, which consists of multiple microbes that can each perform one step in metabolizing an initial resource to a final product when resource inputs are constant [31]. During the obtainment of CON, the fact that the resource input was not constant and the sequential transfers of the enrichment cultures occurred every 7 days [17], which is the point in phenanthrene degradation where the yellow colonies are at their highest value (Fig 2C), could have contributed to the coexistence of the generalist and the resulting processing chain. Temporal oscillations in resource availability, among other environmental dynamics, can create new habitats that allow otherwise competing genotypes to coexist; if conditions oscillate over time, then neither genotype may be able to completely displace the other [28]. Stump and colleagues [31] reported that competition between a generalist and the processing chain may not maximize final compound production, which in our case, could be phenanthrene metabolites and hence phenanthrene degradation (Fig 1A and 1B).

PAH degradation genes were found in the scaffold obtained from CON by the functional metagenomics approach, and S1P3 showed a significant similarity with a genome fragment from *Burkholderia* sp. HB1 chromosome 1 is a phenanthrene-degrading bacteria isolated from a soil sample [35] (Table 1 and S2 Table). Therefore, functional metagenomic results demonstrate the presence of at least another microorganism in CON capable of degrading PAH through the meta-cleavage pathway (Fig 3 and Table 1) related to *Burkholderiales* order. *Betaproteobacteria* class that was previously discovered to be part of CON by pyrosequencing PCR-amplified bacterial 16S rRNA gene fragments from total DNA [18]; *Sphingobium* sp. AM strain, which was found by analyzing its genome, was capable of using the meta- or ortho-cleavage pathway for phenanthrene degradation, as has been reported in other Sphingomonads [36]. Consequently, results suggest that in the studied phenanthrene-degrading natural consortium, there are two major populations that could occupy the same ecological niche. Previous works have demonstrated a synergic interaction between *Sphingomonadales* and *Burkholderiales* orders during PAH degradation in liquid media [37,38]. However, Joshi and co-workers [39] reported that the bioaugmentation of penthachlorophenol-contaminated water with a mixed culture of *Sphingobium chlorophenolicum* and *Burkholderia cepacia* was not as effective as inoculation with pure cultures of both strains, which suggests an antagonistic or competitive effect. The presence of antagonistic relationships in a mixed microbial population, as observed in our work, may exert a negative impact on the ability of contaminant degradation in liquid systems [40]. The interactions themselves between microbial assemblages are context dependent and can readily change over space and time [28].

*Burkholderiales’* ability to degrade aromatic compounds has been previously studied [4,41–44]. Its nutritional versatility and ability to use a wide range of organic compounds as carbon sources has led to the use of *Burkholderia* strains for biodegradation of environmental pollutants [45]. The overall distribution of catabolic genes in the *Burkholderiales* order is more heterogeneous than in other bacterial orders, which might reflect the ecological diversity of this group. These multiple examples consolidate *Burkholderiales* as a main player in the microbial ecology of bioremediation treatments for aromatic decontamination [46]. The complete gene cluster found by metagenomic analysis related to one of the lower pathways involved in PAH degradation (ORFs 31–43) suggests that microorganisms from CON carrying S1P3 DNA fragment could completely degrade catechol via the meta-cleavage pathway (Table 1, Figs 3 and 4). Although ORFs 02, 23, 25 and 30 were located near other degrading genes, all of the putative genes needed for the entire upper pathway were not detected in S1P3, which suggests that they could be distributed along the bacterial genome. The degradation of aromatic compounds through the concerted action of various fragmented pathways has been previously observed in isolated strains and metagenomic fragments [47,48].

The structural configuration of substrates and preferred oxygenation site(s) were the basis for the classification of Ring-Hydroxylanting dioxygenase α-subunits [49]. Using a metagenomic and a metaproteomic approach, it was possible to identify different classes (A, B and C) of dioxygenase that belong to *Sphigomonadales* and *Burkorlderiales* populations of CON. RHO class A preferentially dioxygenates alpha and beta positions of the aromatic ring with respect to the adjacent fused aromatic ring [50], which suggests that the entire protein from ORF 01 in S1P3 could be involved in the first step of PAH biodegradation and also a dioxygenase (present in four copies) in *Sphingobium* sp. AM genome.

Considering the metagenomic results, the fact that in CON the yellow colonies decrease after 7 days of incubation (Fig 2C) and that phenanthrene degradation remained constant at the same point of the incubation time (Fig 1A), it could be argued that in CON, the strain belonging to *Burkholderiales* order is involved in the first steps of the phenanthrene degradation pathway. Stump and co-workers [31] suggested that it is relatively easy for early members in a processing chain to outcompete a generalist but that it is hard for later members of the processing chain. The generalist (AM strain in this work) has a natural advantage over the later members of a processing chain because it can consume earlier resources that the syntrophic species cannot. This advantage becomes greater in each step in the processing chain [31].

In the PAH-degrading strain *Burkholderia artisoli* RP007, the degradation of phenanthrene and naphthalene is supposed to occur via salicylate by the meta-pathway [51,52]. Laurie and co-workers [51] identified a 2433-bp genome fragment from RP007 strain carrying a catechol 2,3-dioxygenase gene preceded by a ferredoxin and followed by a putative 2-hydroxymuconic semialdehyde dehydrogenase. This gene arrangement was also observed in *Burkholderia* sp. HB1 chromosome 1 and scaffold S1P3 (Fig 3, Table 1), but in S1P3, only one ORF (ORF 47) encoding a transcriptional regulator related to GntR proteins was detected upstream from these genes. In general, the GntR family members that control the degradation of aromatic compounds are transcriptional repressors in the absence of the pathway substrates; however, in the presence of the aromatic compound or one of its metabolites, the repression is released [53]. Microorganisms carrying S1P3 DNA fragment probably control the meta-cleavage pathway by a similar mechanism; however, the detailed genetic control of this process is still unknown.

*Sphingobium* sp. AM genome has been previously sequenced and annotated [19], and sequence analysis revealed that it codified for a complete PAH degradation pathway. However, the arrangement of degradative genes in sphingomonads is complex (a characteristic also found in *Sphingobium* sp. AM) with genes scattered across several gene clusters in contrast to the coordinately regulated organized operonic structure of genes in *Burkholderia*, *Pseudomonas* and *Rhodococcus* [49], where genes coding for the meta-cleavage of aromatic rings are largely located on plasmids including promiscuous plasmids pWWO and pTOL [54]. In addition, several genes are a part of, or flanked by, transposon or transposon-like sequences, which suggests that PAH degradation genes are able to spread rapidly within bacterial communities by horizontal gene transfer [55]. The sphingomonads utilize a mechanism of adaptability called ‘flexible’ gene organization, which aids in quick and efficient adjustment to novel compounds in contaminated terrestrial sites [36]. Based on the prediction of regulation, it has already been suggested that multiple inducers are required for the expression of aromatic catabolic enzymes in sphingomonads. The regulation of genes for various aromatic degradation in sphingomonads is quite complex [49].

In concordance with the metagenomic results, the metaproteomic approach showed the expression of proteins belonging to *Shingomonadales* and *Burkholderiales* orders (Table 2). A huge part of the analyzed spots could not be identified, and it could be due to the strong dependence of the proteomics analyses on the size and quality of the reference protein databases against which MS and/or MS/MS data have to be searched [56]. Within the identified proteins GroEL, GroES (both found at days 4 and 15 of incubation and related to *Sphingomonadales* and *Burkholderiales* orders) and aconitate hydratase (found in both analyzed days of incubation and related to *Sphingobium chlorophenolicum* L-1) were reported in previous proteomic studies from PAH-contaminated soils [57,58]. Both chaperonins are known for their role in proper folding of proteins and upregulation in response to toluene and other solvents that dissolve cellular membranes and disrupt protein function [58]. GroEL was also found in the presence of 4-chlorobiphenyl and biphenyl, which demonstrated the stressful conditions these compounds represent to bacteria [59]. A glutathione-S-transferase was also identified in the metaproteomic analysis of CON (Table 2) (found in both analyzed days of incubation and related to *Sphingomonadaceae* family), and these kinds of enzymes are important for the adaptation to oxidative stress due to high reactive oxygen species concentrations generated by the presence of toxic organic compounds [60].

Despite 33% of the identified proteins belonging to *Burkholderiales* order, none were involved in the hydrocarbon metabolic pathway (Table 2). On the contrary, for *Shingomonadales* order, the anthranilate 1,2-dioxygenase small subunit and a toluate/benzoate dioxygenase small subunit (XylY) were found. The low quantity of enzymes directly involved in the PAH degradation pathways could be due to the conducted analysis since only differential spots were analyzed in both conditions.

Through the use of culture technique, we were able to identify only one PAH-degrading strain (belonging to *Sphingomonadales* order), and with a metagenomic approach, the presence of at least one other microorganism in CON capable of degrading PAH (belonging to *Burkholderiales* order). The metaproteomic results indicated that both mentioned orders were metabolically active in CON. The use of proteins could reveal the identity of the active microorganisms and reflect the actual functionality with respect to metabolic reactions and regulatory cascades. Thus, the presence of specific proteins in a sample is a potentially reliable indicator of microbial function [61].

From a technological point of view, the conservation and culture of single strains and as a consequence the construction of a synthetic consortium represent an important advantage with respect to natural consortia.

In some cases, a single generalist is expected to be a better performer than a syntrophic processing chain [31]; however, in this work, the processing chain with only one microorganism with the capability of degrading phenanthrene (synthetic consortium) was more efficient at contaminant and intermediate metabolite degradation than the generalist (*Sphingobium* sp. AM).

## Supporting information

S1 Table Functional assignment of the identified coding sequences found in S1P3 using BLASTp. Nssf: No significant similarity found.
(DOC)

S2 Table S1P3 ORF comparison with the codified proteins in *Burkholderia* HB1. (DOC)
