# Supplementary material for: Assigning ecological roles to the populations belonging to a phenanthrene-degrading bacterial consortium using omic approaches
Source: PLoS One. 2017 Sep 8;12(9):e0184505. doi: 10.1371/journal.pone.0184505 (PMC5591006; doi:10.1371/journal.pone.0184505)
Supplement: S1 Table — Nssf: No significant similarity found. (DOC) [file pone.0184505.s001.doc]

**Supporting information**

**S1 Table.**

| **CDS** | **Blastp (S1P3)** | **%ID** | **%Cov** | **Accesion number** |
| --- | --- | --- | --- | --- |
|
| 1 | dioxygenase alpha subunit DbtAc [Burkholderia fungorum] | 100 | 95 | AGN90996 |
| 2 | 2-hydroxychromene-2-carboxylate isomerase [Burkholderia sp. K24] | 99 | 100 | [WP_051743458.1](http://www.ncbi.nlm.nih.gov/protein/917136746?report=genbank&log$=prottop&blast_rank=1&RID=Z33ZFCJR014) |
| 3 | Nssf | _ | _ | _ |
| 4 | Nssf | _ | _ | _ |
| 5 | Nssf | _ | _ | _ |
| 6 | Nssf |  |  | _ |
| 7 | Fis family transcriptional regulator [Burkholderia sp. K24] | 99 | 99 | WP_030101497.1 |
| 8 | Nssf |  |  | _ |
| 9 | Nssf |  |  | _ |
| 10 | Nssf |  |  | _ |
| 11 | monooxygenase [Rhodocyclaceae bacterium PG1-Ca6] | 74 | 100 | AJP47767.1 |
| 12 | Nssf |  |  | _ |
| 13 | hypothetical protein [Burkholderia sp. K24] | 100 | 100 | WP_030101494.1 |
| 14 | MULTISPECIES:dihydrodipicolinate synthase family protein [Burkholderia] | 99 | 100 | WP_030101493.1 |
| 15 | Nssf |  |  | _ |
| 16 | MULTISPECIES: 4-hydroxythreonine-4-phosphate dehydrogenase [Burkholderia] | 99 | 100 | WP_030101492 |
| 17 | MULTISPECIES: hypothetical protein [Burkholderia] | 100 | 100 | WP_030101491.1 |
| 18 | MULTISPECIES: benzene 1,2-dioxygenase [Burkholderia] | 99 | 100 | WP_030101490.1 |
| 19 | MULTISPECIES: hypothetical protein [Burkholderia] | 99 | 100 | WP_030101489.1 |
| 20 | hypothetical protein [Burkholderia sp. K24] | 99 | 100 | WP_030101488.1 |
| 21 | Rieske (2Fe-2S) domain-containing protein [Burkholderia sp. Ch1-1] | 99 | 100 | EIF28468.1 |
| 22 | long-chain fatty acid transport protein [Burkholderia sp. Ch1-1] | 99 | 100 | EIF28467 |
| 23 | MULTISPECIES: aldolase [Burkholderia] | 100 | 100 | WP_007179229 |
| 24 | ferredoxin reductase DbtAa [Burkholderia fungorum] | 100 | 100 | AGN90994 |
| 25 | putative hydrolase [Burkholderia sp. DBT1] | 99 | 100 | AAK96192 |
| 26 | putative monoxygenase alpha subunit [Burkholderia sp. DBT1] | 99 | 100 | AAK96191 |
| 27 | ferrodoxin DbtAb [Burkholderia fungorum] | 100 | 100 | AGN90995 (DBT1 AAK96190) |
| 28 | extradiol dioxygenase DbtC [Burkholderia sp. DBT1] | 99 | 100 | AAK96189 |
| 29 | putative oxidoreductase [Burkholderia sp. DBT1] | 99 | 100 | AAK96188 |
| 30 | putative isomerase [Burkholderia sp. DBT1] | 98 | 100 | AAK96187 |
