# Supplementary material for: Assigning ecological roles to the populations belonging to a phenanthrene-degrading bacterial consortium using omic approaches
Source: PLoS One. 2017 Sep 8;12(9):e0184505. doi: 10.1371/journal.pone.0184505 (PMC5591006; doi:10.1371/journal.pone.0184505)
Supplement: S2 Table — (DOC) [file pone.0184505.s002.doc]

**Supporting information**

**S2 Table.**

| **ORF** | **Start** | **Stop** | **Strand** | **Function** | **Blastp (% id)** | **Accesion Number** |  |
| --- | --- | --- | --- | --- | --- | --- | --- |
| 14 | 7618 | 6656 | - | 4-hydroxy-tetrahydrodipicolinate synthase (EC 4.3.3.7) | 99 | WP_030101493.1 |  |
| 15 | 7687 | 7884 | + | hypothetical protein |  |  |  |
| 16 | 9056 | 8037 | - | 4-hydroxythreonine-4-phosphate dehydrogenase (EC 1.1.1.262) | 99 | WP_030101492 |  |
| 17 | 9582 | 9073 | - | Benzoate 1,2-dioxygenase beta subunit (EC 1.14.12.10) | 100 | WP_030101491.1 |  |
| 18 | 10955 | 9588 | - | Benzoate 1,2-dioxygenase alpha subunit (EC 1.14.12.10) | 99 | WP_030101490.1 |  |
| 19 | 12668 | 11055 | - | Choline dehydrogenase (EC 1.1.99.1) | 99 | WP_030101489.1 |  |
| 20 | 13205 | 12720 | - | Ortho-halobenzoate 1,2-dioxygenase beta-ISP protein OhbA | 100 | WP_053858387 |  |
| 21 | 14413 | 13208 | - | Large subunit naph/bph dioxygenase | 99 | WP_030101487 |  |
| 22 | 15842 | 14508 | - | Long-chain fatty acid transport protein | _ | _ |  |
| 23 | 17441 | 16440 | - | 4-hydroxy-tetrahydrodipicolinate synthase (EC 4.3.3.7) | 100 | WP_007179229.1 |  |
| 24 | 18481 | 17462 | - | Toluene-4-monooxygenase, subunit TmoF | 100 | WP_053858388.1 |  |
| 25 | 19321 | 18491 | - | 2-hydroxy-6-oxo-6-phenylhexa-2,4-dienoate hydrolase (EC 3.7.1.-) | 99 | WP_051743457.1 |  |
| 26 | 20596 | 19376 | - | luciferase family protein | 100 | WP_035519965.1 |  |
| 27 | 20962 | 20651 | - | Ferredoxin subunits of nitrite reductase and ring-hydroxylating dioxygenases | _ | _ |  |
| 28 | 21950 | 21036 | - | 2,3-dihydroxybiphenyl 1,2-dioxygenase (EC 1.13.11.39) | 99 | WP_030101481.1 |  |
| 29 | 23915 | 22926 | - | Quinone oxidoreductase (EC 1.6.5.5) | 99 | WP_053858389.1 |  |
| 30 | 24741 | 24151 | - | 2-hydroxychromene-2-carboxylate isomerase | 97 | WP_030101479.1 |  |
| 31 | 26023 | 24755 | - | Large subunit toluate/benzoate dioxygenase | 98 | WP_053858390.1 |  |
| 32 | 26522 | 26034 | - | Ortho-halobenzoate 1,2-dioxygenase beta-ISP protein OhbA | 100 | WP_030101477.1 |  |
| 33 | 27464 | 26679 | - | 1,2-dihydroxycyclohexa-3,5-diene-1-carboxylate dehydrogenase (EC 1.3.1.25) | 98 | WP_030101476.1 |  |
| 34 | 28310 | 27474 | - | 2-hydroxymuconic semialdehyde hydrolase (EC 3.7.1.9) | 97 | WP_030101475.1 |  |
| 35 | 28985 | 28374 | - | Glutathione S-transferase (EC 2.5.1.18) | 99 | WP_030101474.1 |  |
| 36 | 29262 | 29071 | - | 4-oxalocrotonate tautomerase (EC 5.3.2.-) | 100 | WP_030101473.1 |  |
| 37 | 30079 | 29291 | - | 4-oxalocrotonate decarboxylase (EC 4.1.1.77) | 98 | WP_030101472.1 |  |
| 38 | 31121 | 30084 | - | 4-hydroxy-2-oxovalerate aldolase (EC 4.1.3.39) | 99 | WP_053858391.1 |  |
| 39 | 32041 | 31121 | - | Acetaldehyde dehydrogenase, acetylating, (EC 1.2.1.10) | 98 | WP_030101470.1 |  |
| 40 | 32825 | 32061 | - | 4-oxalocrotonate decarboxylase (EC 4.1.1.77) | 100 | WP_053858392.1 |  |
| 41 | 34302 | 32848 | - | Putative 5-carboxymethyl-2-hydroxymuconate semialdehyde dehydrogenase oxidoreductase protein (EC 1.2.1.60) | 99 | WP_030101468.1 |  |
| 42 | 35475 | 34525 | - | Catechol 2,3-dioxygenase (EC 1.13.11.2) | 99 | WP_030101467.1 |  |
| 43 | 35969 | 35559 | - | Iron-sulfur binding electron transfer protein | 100 | WP_030101466.1 |  |
| 44 | 36448 | 36573 | + | hypothetical protein |  |  |  |
| 45 | 36570 | 36689 | + | hypothetical protein |  |  |  |
| 46 | 36820 | 36686 | - | hypothetical protein |  |  |  |
| 47 | 38011 | 37223 | - | Regulatory protein GntR, HTH:GntR, C-terminal |  |  |  |
| 48 | 38270 | 38704 | + | Mobile element protein |  |  |  |
| 49 | 38734 | 39255 | + | Mobile element protein |  |  |  |
